# Clinical decision-making frameworks for pandemic preparedness and response: A scoping review

**DOI:** 10.34172/hpp.025.44995

**Published:** 2025-12-30

**Authors:** Farhad Sattar Mohammed, Kaveh Bahmanpour, Sina Valiee, Adel Fatemi

**Affiliations:** ^1^Department of Health Care Management, School of Medical Sciences and Technologies, S R.C., Islamic Azad University, Tehran, Iran; ^2^Department of Nursing, School of Medical Sciences, Sa.C., Islamic Azad University, Sanandaj, Iran; ^3^Clinical Care Research Center, Research Institute for Health Development, Kurdistan University of Medical Sciences, Sanandaj, Iran; ^4^Department of Statistics, School of Basic Sciences, Sa.C., Islamic Azad University, Sanandaj, Iran

**Keywords:** Clinical decision making, Pandemics, Emergency preparedness, Scoping review

## Abstract

**Background:**

Pandemic management demands a multifaceted strategy that integrates disease transmission control, resource allocation, and effective public health interventions. This study explores how combining clinical decision-making frameworks (CDMFs) can improve decision-making and adaptive strategies during public health emergencies. The goal is to provide a synergistic approach that enhances the speed, effectiveness, and equity of pandemic responses.

**Methods:**

We conducted a review of literature published up to January 2025 to evaluate the contributions and limitations of these frameworks in pandemic preparedness and response. The review emphasizes how each framework supports adaptability, risk identification, and strategic planning, while also addressing challenges related to equity and data quality.

**Results:**

The SOAR framework fosters adaptability and creativity, while risk assessment provides a systematic method for threat identification and mitigation. Artificial intelligence (AI)-driven decision support system (DSS) leverage machine learning and predictive analytics to provide immediate insights and improve strategic planning, although issues of data quality and equity must be addressed. The DECIDE framework ensures comprehensive decision-making, balancing strategic planning with the urgency of a crisis. The review highlights the potential of AI to improve decision-making efficiency, while underscoring the need for careful oversight to maintain transparency and prevent the perpetuation of health inequalities.

**Conclusion:**

Integrating AI into CDMFs offers significant opportunities to improve future pandemic responses. Evolving these frameworks and incorporating AI-DSS, while carefully addressing ethical considerations and data quality, will lead to more scientifically sound, practical, and equitable solutions to global health problems, enhancing overall pandemic preparedness and resilience.

## Introduction

 Global health security is continually challenged by the emergence and re-emergence of infectious diseases, with pandemics posing particularly acute threats that can overwhelm healthcare systems and strain the boundaries of medical readiness. Notable outbreaks, including the 2009 H1N1 influenza pandemic, the 2014–2016 Ebola epidemic in West Africa, and the ongoing COVID-19 pandemic caused by SARS-CoV-2, have exposed critical vulnerabilities in health infrastructures worldwide.^1,2^ A central challenge in such contexts is the imperative for clinicians to make rapid, high-stakes decisions under conditions marked by profound uncertainty, rapidly evolving evidence, resource constraints, and surges in patient volume.^3,4^

 Under routine circumstances, clinical decision-making is typically guided by robust evidence derived from randomized controlled trials, established protocols, and multidisciplinary consultations. Pandemics, however, disrupt this normative paradigm. A confluence of factors—including overwhelming patient influx, initial absence of effective therapeutics, constrained diagnostic capabilities, and critical shortages of resources such as personal protective equipment (PPE), ventilators, and staffing—creates an environment in which conventional practice standards become inadequate.^5^ This shift necessitates a transition from individualized patient-centered care toward a population health approach, wherein the objective is to maximize benefits for the largest number of people.^6^ In such settings, structured frameworks become indispensable for guiding triage, resource allocation, and treatment protocols in a consistent, ethically sound, and equitable manner.

 Evidence-based decision-making (EBDM) serves as a cornerstone of pandemic response, leveraging the best available scientific evidence to inform critical actions. During outbreaks such as H1N1, SARS, and COVID-19, EBDM has guided resource allocation, containment strategies, and public health communications through predictive modeling, surveillance, and the development of clinical guidelines.^7-9^ However, the dynamic and uncertain nature of pandemics often strains traditional EBDM processes, creating a pressing need to integrate real-time data analytics and artificial intelligence (AI) to enhance predictive accuracy when information is incomplete or evolving rapidly.^7,8^

 Clinical decision-making frameworks (CDMFs) for pandemics are designed to provide precisely this structure. These systematic tools integrate empirical evidence, ethical principles, and operational pragmatics to assist healthcare providers in making consistent, transparent, and justifiable decisions. CDMFs may address a range of critical issues, including prioritization for intensive care, allocation of scarce interventions (e.g., ventilators, antivirals, monoclonal antibodies), and modifications to elective surgical and procedural care.^10,11^ The formulation of these frameworks typically involves multidisciplinary collaboration among clinicians, ethicists, public health officials, and hospital administrators to ensure that they are both clinically appropriate and logistically viable.^12^

 Several specialized frameworks complement EBDM by addressing specific aspects of pandemic decision-making. The Ottawa Decision Support Framework (ODSF), for instance, focuses on the process of decision-making at both individual and community levels.^13^ Although originally designed for shared clinical decisions, its principles—evaluating decisional needs, providing tailored support, and assessing outcomes—are vital for fostering public trust and adherence during a crisis.^13-15^ The PRECIS-2 tool (Pragmatic Explanatory Continuum Indicator Summary-2) aids in designing clinical trials that are fit for purpose during emergencies, promoting pragmatic studies that generate results directly applicable to real-world settings, thereby accelerating the evaluation of novel therapies and vaccines.^16-19^

 Despite their recognized importance, the extant literature on pandemic CDMFs is fragmented. Multiple institutions, governmental bodies, and professional societies have put forward various guidelines and models, yet these vary considerably in quality, scope, methodological rigor, and practical applicability.^20^ While some are grounded in well-defined ethical reasoning, others function primarily as operational checklists. Moreover, the urgency driving the development of such frameworks during recent crises has prompted questions regarding their implementability, effectiveness in improving patient outcomes, and integration of lessons learned from prior pandemics.^21^

 Thus, a synthesis of the available evidence is essential to map, evaluate, and consolidate knowledge on CDMFs for pandemic preparedness and response. This scoping review aims to identify, critically appraise, and summarize the characteristics, core components, and methodological foundations of published CDMFs intended for use in pandemics. The findings of this review will provide an evidence base to support the development, refinement, and implementation of robust decision-making tools for future public health emergencies.

## Materials and Methods

 This scoping review was conducted to synthesize the available literature on CDMFs for pandemic preparedness and response published between January 2000 and January 2025. We tried to design and report the review in accordance with the Preferred Reporting Items for Systematic Reviews and Meta-Analyses (PRISMA) guidelines.

###  Search strategy

 A comprehensive search was performed across four electronic databases: PubMed, Scopus, Web of Science, and Google Scholar. The search strategy was developed using a combination of controlled vocabulary (e.g., MeSH terms) and keywords related to clinical decision-making, pandemics, and response frameworks. Key search terms included: “clinical decision-making framework,” “pandemic preparedness,” “pandemic response,” “decision support systems,” “artificial intelligence,” “predictive modeling,” and “crisis standards of care.” Boolean operators (AND, OR) were employed to refine the search. The full search strategy for each database is provided in Table 1. To minimize the risk of publication bias, additional sources were identified through manual screening of reference lists of included articles and relevant review papers. Grey literature, including technical reports, guidelines from governmental and non-governmental organizations (e.g., WHO, CDC), and preprints, was also considered for inclusion.

**Table 1:** Inclusion and exclusion criteria, search terms and databases

**Items **	**Explanation **
Search strategy & syntaxes	A systematic search was conducted in PubMed, Google Scholar, Scopus, and Web of Science using the following search syntax: - ("clinical decision-making frameworks" OR "healthcare decision support systems") AND ("pandemic preparedness" OR "pandemic response") - ("AI in healthcare decision-making" OR "predictive modeling in pandemics").
Date of search	Searches were performed from January 2000 to January 2025.
Keywords	Clinical decision-making frameworks, pandemic preparedness, pandemic response, healthcare decision support systems, AI in healthcare decision-making, predictive modeling in pandemics.
Inclusion criteria	-Empirical or theoretical studies focused on clinical decision-making frameworks applied to pandemic situations. -Studies evaluating decision support tools, predictive models, or frameworks during pandemic responses (e.g., SARS, H1N1, COVID-19). -Research providing insights into the development or assessment of such frameworks. -Grey literature (e.g., government or WHO reports) discussing frameworks, best practices, or lessons learned in pandemic management. -Published in English.

###  Eligibility criteria

 Eligibility for inclusion required that studies focus on the development, application, or evaluation of CDMFs or tools within pandemic contexts, be published between 2000 and 2025 (January), and be available in English; both empirical investigations—such as cohort studies, case studies, and randomized trials—and conceptual or theoretical articles were considered, in addition to grey literature that offered substantive insights into framework design or implementation, while exclusions encompassed studies not specific to pandemics (e.g., those addressing natural disasters or isolated outbreaks), non-peer-reviewed articles (unless from an authoritative source and presenting unique data), and publications lacking a clear focus on decision-making frameworks.

###  Study selection and data extraction

 Study selection was conducted by two independent reviewers through a two-stage process involving initial title and abstract screening followed by a comprehensive full-text assessment, with any discrepancies resolved via consensus or arbitration by a third reviewer; data extraction was performed using a standardized, piloted form to capture study characteristics (e.g., author, year, country, design), framework details (including name, objectives, and components), pandemic context (such as COVID-19 or H1N1), relevant stakeholders, reported outcomes (e.g., efficacy, usability, limitations), and key findings and implications.

###  Quality assessment

 Methodological quality of included studies was assessed using the Joanna Briggs Institute (JBI) Critical Appraisal Tools,^22,23^ appropriate to study design. Randomized controlled trials were evaluated for randomization method, blinding, and attrition bias. Observational studies were appraised based on sampling strategy, comparability, and outcome measurement. Theoretical articles and grey literature were assessed for clarity, logical consistency, and relevance. Each study was rated as having high, moderate, or low quality. Two reviewers independently performed the assessments, with conflicts resolved by consensus or third-reviewer arbitration.

###  Data synthesis

 Given the anticipated heterogeneity among frameworks and outcomes, a narrative synthesis was conducted, whereby extracted data were organized thematically according to framework type (such as AI-driven, ethics-guided, or operational), application context (including triage and resource allocation), reported strengths and limitations, and lessons derived from real-world implementation; the findings were subsequently summarized to identify recurring patterns, evidence gaps, and potential directions for future research and development.

## Results

**Figure 1:**
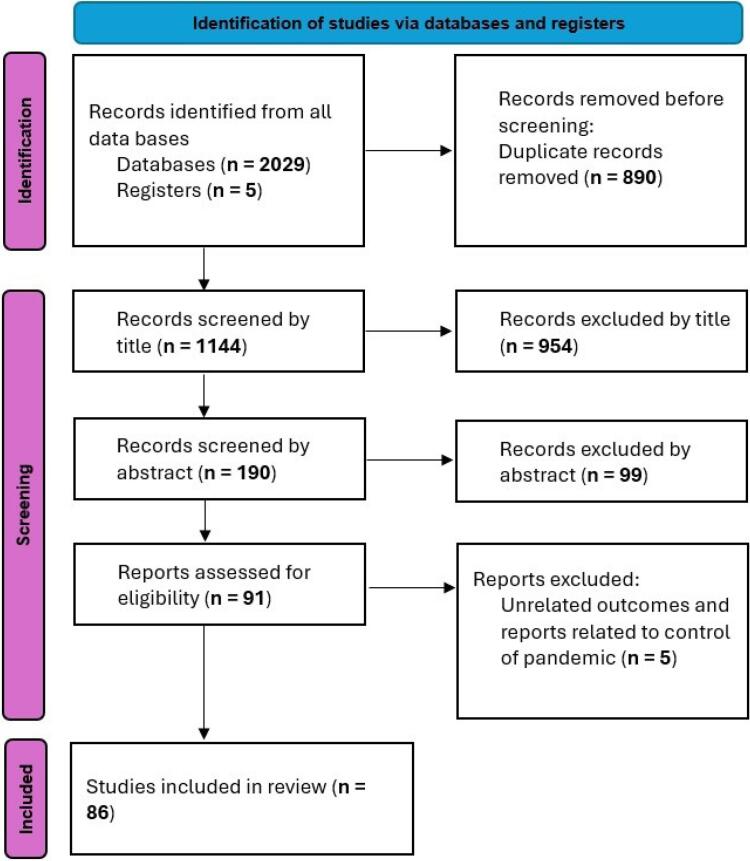
A flow diagram of study identification, screening, and inclusion

 Our search and selection process yielded 86 studies for inclusion in this review. The identification of records is summarized in Figure 1. From an initial pool of 2,034 records identified from databases and registers, 890 duplicates were removed. Screening of 1,144 titles and 190 abstracts excluded 954 and 99 records, respectively, for irrelevance. Of the 91 full-text articles assessed for eligibility, 5 were excluded as they focused on pandemic management outcomes rather than decision-making frameworks, resulting in the final 86 studies included.

**Figure 2:**
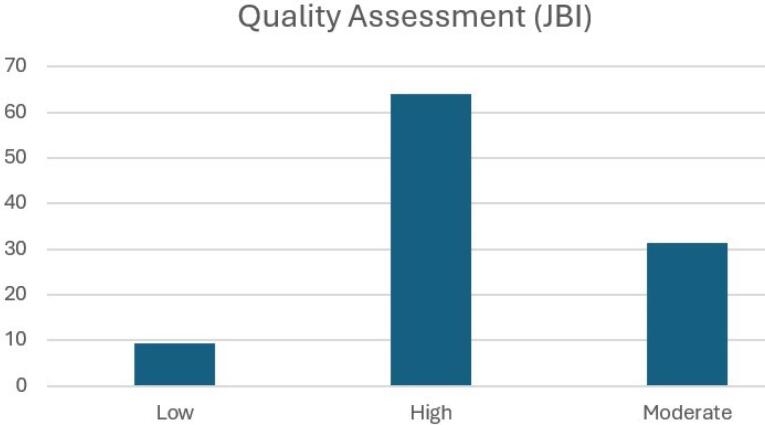
Quality assessment of included studies, based on the JBI Critical Appraisal Tool

 The quality assessment of these studies, performed using the JBI Critical Appraisal Tools, indicated that the majority (63.95%) were of high quality with minimal risk of bias. A significant portion (31.40%) was of moderate quality, offering valuable evidence despite some methodological limitations. A small percentage (9.3%) were classified as low quality but were retained due to their unique insights into the application of the frameworks under review (Figure 2).

 The findings from the included studies are synthesized below, highlighting the core characteristics, pandemic applications, strengths, and limitations of each CDMF. A comprehensive summary of these results is presented in Table S1 (see Supplementary file 1).

###  Evidence-based decision-making 

 EBDM emerged as a foundational approach, leveraging predictive modeling, surveillance, guideline development, and communication to ground pandemic responses in scientific evidence.^24,25^ Predictive models, such as the Susceptible-Infected-Recovered (SIR) framework, were instrumental during the H1N1 and COVID-19 pandemics for forecasting disease spread and informing resource allocation and containment strategies.^26,27^ Surveillance systems, notably the WHO’s Global Influenza Surveillance and Response System (GISRS), enhanced early detection capabilities. The integration of big data and AI further improved real-time outbreak monitoring. However, the efficacy of EBDM was frequently challenged by the rapid evolution of pandemics, often leading to decisions based on incomplete data. Significant barriers were noted in resource-constrained settings, where disparities in data integration and coordination hampered response efforts.^27-29^ The continuous updating of guidelines by bodies like the CDC and WHO, alongside communication strategies that managed misinformation and promoted cultural sensitivity—as demonstrated during the Ebola outbreak—were critical for maintaining public trust and standardizing care.^25,29-31^ Effective EBDM was found to necessitate robust interdisciplinary collaboration across epidemiology, data science, and public health policy.^28,29,31^

## The Ottawa Decision Support Framework (ODSF)

 The ODSF provided a structured, adaptable process for supporting decisions at individual and community levels, which proved vital for maintaining public trust and compliance.^32-34^ Its application during the COVID-19 pandemic involved assessing public perceptions of risk and decisional needs regarding measures like masking and vaccination, thereby refining tailored communication strategies.^15,35-37^ The framework empowered individuals through accessible tools and guidance and ensured consistency in public health interventions by training healthcare workers and community leaders.^38-40^ A key strength was its utility in evaluating policy effectiveness by assessing alignment with health outcomes, compliance rates, and broader impacts on mental health and social cohesion. However, the process of assessing decisional needs was reported as time-intensive and less practical for large-scale emergencies. Challenges also included ensuring the framework’s adaptability for quick updates in fast-evolving scenarios,^39^ its scalability from individual to national levels, and the necessity for cultural sensitivity to address diverse population needs.^38-40^

###  Pragmatic Explanatory Continuum Indicator Summary-2 

 The PRECIS-2 framework was identified as a key tool for enhancing the real-world applicability and relevance of clinical research during health crises.^19,41-43^ It facilitates the design of pragmatic trials that prioritize inclusivity, rapid enrollment, and generalizable results—critical needs during a pandemic.^16,44,45^ This is achieved by broadening eligibility criteria, employing flexible recruitment strategies, utilizing real-world settings like community health centers, and adapting delivery and adherence monitoring to fluctuating resources.^16-18,44,45^ The framework minimizes strain on healthcare systems by leveraging existing health records for follow-up and focusing analysis on critical outcomes like mortality and hospitalization rates. Its main limitation is the requirement for significant expertise in trial design, which can restrict its usability for non-research professionals, and its feasibility can be limited in severely resource-constrained environments.^46^

###  Health technology assessment (HTA)

 HTA was critical for the rapid and equitable evaluation of new technologies and interventions during pandemics. By assessing cost-effectiveness, societal value, and equity, HTA guides optimal resource allocation, ensuring that the most beneficial technologies are prioritized when resources are scarce.^47-49^ Accelerated processes that incorporate real-world evidence enable timely decision-making in crisis situations. However, the traditional HTA process is inherently time-consuming, limiting its agility and responsiveness in rapidly changing scenarios.^49-52^ Effective HTA requires interdisciplinary collaboration to fully assess a technology’s impact, but its pace can be a mismatch for the urgent demands of a pandemic, necessitating more streamlined or adaptive methods.^49-52^

###  SOAR framework

 The SOAR framework offered a positive, strengths-based approach to strategic planning, focusing on an organization’s Strengths, Opportunities, Aspirations, and Results. In a pandemic context, this focus helps organizations leverage existing capabilities (e.g., skilled workforce, technology) to meet pressing demands,^53-55^ identify opportunities for innovation (e.g., digital health solutions), and align efforts with aspirational goals like equitable care.^54,56^ This approach fosters resilience, motivation, and measurable accountability.^56^ A notable limitation is its potential to overlook immediate weaknesses and threats, such as resource constraints or system failures, which are critical in a crisis and are typically addressed by more comprehensive frameworks like SWOT.^54^

###  Risk assessment and management framework

 These frameworks provided a structured methodology for identifying, analyzing, mitigating, and monitoring risks throughout a pandemic.^57^ They enable timely interventions through early threat detection, support dynamic adaptation via continuous monitoring, and facilitate public compliance through clear risk communication.^58-60^ Their effectiveness, however, is highly dependent on the availability of accurate and timely data, which is often scarce in the early stages of an outbreak.^57,61-64^ Resource constraints can further limit the implementation of optimal risk mitigation strategies, highlighting the need for approaches that are both adaptive and responsive to evolving conditions.^57,63,64^

## AI-driven decision support systems (DSS)

 AI-driven DSS emerged as powerful tools for enhancing decision-making by processing vast amounts of data to provide real-time insights, predict trends, and optimize resources.^65,66^ These systems integrate diverse data sources, use predictive modeling for forecasting, and offer tools for scenario planning and resource allocation.^67^ User-friendly dashboards visualize key metrics, and AI can enhance communication through automated reporting and culturally sensitive messaging. A significant strength is their capacity for continuous learning, improving predictions over time.^68-70^ However, their performance is contingent on high-quality data; poor data or flawed model assumptions can lead to inaccurate outputs. Key challenges include ensuring data privacy, maintaining algorithmic transparency, avoiding the perpetuation of biases, and preventing over-reliance on AI without adequate human oversight.^68,69^

###  DECIDE framework

 The DECIDE framework offers a comprehensive, structured process for thorough and actionable decision-making, ideal for strategic planning and policy formulation.^71^ Its steps—defining the problem, establishing criteria, considering alternatives, identifying the best solution, developing an implementation plan, and evaluating outcomes—ensure clarity and reduce bias.^71-73^ While this systematic approach promotes defensible and well-considered decisions, it can be perceived as overly methodical and time-consuming for the rapid decision-making required in a fast-moving pandemic, potentially hindering operational agility.^71,72^

###  Synthesis of framework characteristics

 The reviewed frameworks offer complementary strengths for pandemic management. EBDM, AI-DSS, and Risk Assessment provide the data-driven, evidence-based foundation for response strategies. ODSF and PRECIS-2 focus on the human and practical elements, ensuring stakeholder engagement and real-world applicability. HTA and DECIDE offer structured evaluation and strategic processes for resource allocation and long-term planning. The SOAR framework contributes a positive, forward-looking lens to foster innovation and resilience. A common challenge across nearly all frameworks was the dependency on timely, high-quality data and the need for adaptability in the face of uncertainty and resource constraints.

## Discussion

 The management of pandemics presents significant challenges due to the rapid spread of disease, unpredictability, and the need for informed decision-making. Frameworks such as EBDM, the ODSF, and PRECIS-2 provide structured approaches to address these complexities. EBDM enables proactivity through the use of prediction models, such as the SIR model for H1N1^74^ and COVID-19,^75^ to forecast disease spread and resource needs. For practice, this highlights the need for healthcare systems to invest in and utilize predictive modeling tools to anticipate surges in patient volume and allocate resources effectively. Training personnel in the interpretation and application of these models is also crucial. However, the success of EBDM relies on high-quality, real-time data, which is often difficult to obtain during pandemics.^7^ The integration of AI and big data can enhance this process, but it also brings its own challenges, such as data silos.^76^ Therefore, healthcare organizations should prioritize the development of robust data collection and integration systems, ensuring data quality, standardization, and interoperability.

 The ODSF adds a layer of personalization, which is critical for public engagement and compliance with health measures. By addressing stakeholder needs and providing tailored decision support, the ODSF can improve public trust.^15,35,37^ In practice, this means that public health communications should be tailored to specific communities, addressing their unique concerns and providing clear, actionable guidance. Utilizing community leaders and trusted messengers can enhance the effectiveness of these communications. However, the urgency of decisions and the time required to assess needs can make its use difficult. The PRECIS-2 framework, which advocates pragmatic trials, allows for the rapid generation of evidence, but a balance needs to be struck between scientific rigor and practical implementation in a crisis context.^17,18,45^ For practical application, researchers and funding agencies should prioritize pragmatic trial designs that can be rapidly implemented in real-world settings, focusing on outcomes that are most relevant to patients and healthcare providers.

 To ensure cost-effectiveness and equity under resource constraints, HTA is essential for evaluating pandemic interventions. However, the traditional HTA process is time-consuming, so adaptive methods are essential to enable rapid decision-making.^47,49,52^ In practice, this requires healthcare systems to develop streamlined HTA processes that can quickly assess the value of new technologies and interventions during a pandemic, considering both clinical effectiveness and cost-effectiveness. The SOAR framework encourages innovation and resilience, promoting solutions such as telemedicine or rapid vaccine development by focusing on strengths and aspirations.^47,49,52-54^ Organizations can use the SOAR framework to identify their key strengths and opportunities, fostering innovation and adaptation in the face of challenges. For example, healthcare systems can leverage their existing infrastructure and expertise to rapidly implement telemedicine programs or develop new diagnostic tools. However, it may overlook immediate risks that other frameworks, such as SWOT, address.

 Risk assessment and management frameworks are fundamental to the identification and dynamic management of risk, but their effectiveness is often limited by data availability and the ability to act quickly.^77^ Therefore, healthcare organizations should invest in robust risk assessment and management systems, ensuring that they have access to timely and accurate data, and that they can rapidly implement mitigation strategies when necessary. DSS have the potential to improve decision-making by processing data and predicting outcomes, although concerns remain regarding privacy and transparency.^65,78^ In practice, this means that healthcare systems should carefully evaluate the potential benefits and risks of implementing DSS, ensuring that they are used in a way that is ethical, transparent, and equitable. Finally, the DECIDE framework provides a structured approach that is ideal for strategic pandemic planning. However, it needs to be adapted to allow agility in decision making. It must balance thoroughness with the urgency required in a crisis.^65,71,78^

 By addressing challenges such as data overload, resource optimization, and predictive analysis, the integration of AI-DSS into these frameworks could significantly improve pandemic management. AI-DSS can be an invaluable tool for EBDM by providing rapid insights and predictions, with the potential to process vast amounts of data in real time. AI-DSS has the potential to process vast amounts of data, making it an invaluable tool for EBDM, providing rapid insights and predictions to guide HTA, providing data-driven evaluations of interventions and technology, and supporting the ODSF, providing personalized recommendations to individuals in real-time. For practice, this underscores the importance of investing in AI-DSS and training healthcare professionals to effectively use these tools. AI-DSS can support decision-making at all levels of the healthcare system, from individual patient care to resource allocation and public health policy. However, integrating AI into these frameworks comes with its own challenges. Issues such as data protection, the transparency of AI algorithms, and the risk of perpetuating existing health inequalities all need to be addressed. Therefore, healthcare organizations should develop clear guidelines and protocols for the use of AI in healthcare, ensuring that these tools are used in a way that is ethical, transparent, and equitable. In addition, during a fast-moving pandemic, the ability of AI to provide real-time support is highly dependent on the quality and timeliness of data. Therefore, while there is immense potential for AI to improve the speed and accuracy of decision making, its use will need to be carefully managed to ensure that it remains equitable, transparent and in line with both local needs and global evidence.

## Conclusion

 In summary, effective pandemic management necessitates the integrated application of diverse frameworks, including EBDM, ODSF, and HTA, each offering unique strengths to address multifaceted challenges. While these frameworks provide structure and guidance, their utility is tempered by persistent challenges related to data timeliness, cultural adaptation, and equity. AI-driven DSSs hold substantial promise for enhancing real-time insights and predictive capabilities, yet their deployment raises critical ethical considerations and the potential for exacerbating existing health inequities.

 Moving forward, it is crucial to prioritize the adaptation of these frameworks to accommodate rapidly evolving situations, ensuring that decisions are both scientifically sound and practically implementable. Specifically, healthcare organizations and public health agencies should invest in training programs to equip personnel with the skills to effectively utilize these frameworks and AI-DSS tools. Furthermore, collaborative efforts are needed to establish standardized data collection and sharing protocols, addressing issues of data quality and interoperability. Continued innovation and interdisciplinary collaboration are essential to refine these approaches, fostering resilience and ensuring equitable and effective responses to future pandemics. Therefore, we urge policymakers and researchers to focus on developing pragmatic strategies that bridge the gap between theoretical frameworks and real-world implementation, ultimately safeguarding global health security.

## Data availability statement

 Data are available on reasonable request. The data that support the findings of this research are available from Vice-Chancellor for Research, Science and Research branch, Islamic Azad University, Tehran, Iran but restrictions apply to the availability of these data, which were used under license for the current study, and so are not publicly available. Data are however, available from the authors upon reasonable request and with permission of Vice -Chancellor for Research.

## Competing Interests

 There are no competing interests related to this project.

## Ethical Approval

 Ethics committee approval is not required as it is a review used only secondary data.

## Supplementary Files

Supplementary file 1 contains Table S1.
