# Supplementary material for: Clinical decision-making frameworks for pandemic preparedness and response: A scoping review
Source: Health Promot Perspect. 2025 Dec 30;15(4):333–42. doi: 10.34172/hpp.025.44995 (PMC13156281; doi:10.34172/hpp.025.44995)
Supplement: Supplementary file 1 — contains Table S1. [file hpp-15-333-s001.pdf]

Mohammed et al, **Health Promotion Perspectives.** 2025;15(4):333-S1

doi: 10.34172/hpp.025.44995

https://hpp.tbzmed.ac.ir

Table S1: Core characteristics, pandemic applications, strengths, and limitations of the reviewed clinical decision-making frameworks

Frameworks	Components	Purpose	Applications	Limitations	Strengths
**Evidence-Based Decision-Making (EBDM)**	Predictive Modeling	To anticipate the spread and impact of infectious diseases.	Mathematical models are used to simulate the spread of disease, estimate peak periods and predict healthcare resource needs, such as the Susceptible, Infected, Recovered (SIR) model. Models were instrumental in shaping vaccination and containment strategies during the H1N1 influenza pandemic. More recently, models have played a key role in forecasting the course of COVID-19 and have informed decisions on containment, social distancing and health system readiness.	Challenges in rapidly evolving scenarios with incomplete evidence.	Ensures decisions are grounded in rigorous evidence and adaptable to various healthcare settings.
	Surveillance Systems	Early detection and monitoring of disease patterns.	Advanced monitoring involves syndromic monitoring, sentinel sites and using digital tools for real-time data capture. An example of how continuous monitoring of influenza strains helps to ensure timely responses is WHO's Global Influenza Surveillance and Response System (GISRS). As seen in the use of social media and mobile health data during the pandemic, the integration of big data and AI in surveillance has improved the ability to detect outbreaks early.	Integration of disparate data sources and timeliness in resource-limited settings.	Improves early detection and response capabilities through innovative technology.
	Guideline Development	To provide standardized, evidence-based treatment and prevention protocols.	The development of clinical management guidelines involves synthesizing available evidence into actionable recommendations. During the COVID-19 pandemic, organizations like the CDC and WHO have regularly updated guidelines on everything from testing protocols to patient management, adapting as new evidence emerges. These guidelines are crucial for consistency in clinical practice across different regions.	Delays in updating guidelines in fast-evolving scenarios.	Ensures consistency in clinical practices and adapts to emerging evidence.
	Effective Communication	Provide clear, accurate and timely information to healthcare providers, policy	Communication frameworks have been developed for the management of misinformation, the promotion of public health interventions and the coordination of responses. The importance of community engagement and culturally sensitive communication was highlighted by the success of public health messaging during the Ebola outbreak in West Africa. In the context of pandemics, effective	Difficulty in countering misinformation and maintaining public trust.	Builds public trust and facilitates coordinated responses through

		makers and the public.	communication also includes the management of media relations, the trust of the public and the transparency of the decision-making process.		transparency and engagement.
**Ottawa Decision Support Framework (ODSF)**	**Assessing Decisional Needs**	To understand the specific information and decision-making needs of stakeholders.	To do so, we need to understand their specific information and decision-making needs - from providers to policymakers to citizens. During the COVID-19 crisis, for example, it became necessary to assess individuals' perceptions of risk and the support needed to make decisions on masking, distancing and vaccination.	Time-intensive and less practical for large-scale emergencies.	Provides tailored insights into stakeholder needs.
	**Providing Decision Support**	To help guide individuals through complex decisions with clear, relevant information.	Clinical guidance during pandemics focuses on disseminating accurate, accessible, and audience-specific information through diverse channels. Ensuring clarity and relevance is crucial for effective communication. Decision support tools, such as apps or systems, can guide individuals through complex choices, including quarantine protocols and travel restrictions. Additionally, training health workers and community leaders to assist with decision-making during crises fosters informed choices and minimizes conflict.	Requires resources for implementation and dissemination.	Empowers stakeholders and incorporates their values into decisions.
	**Evaluating Decisional Outcomes**	To evaluate the impact of decisions and policies on health outcomes, compliance, and social factors.	During a pandemic, it is important to assess the alignment of policies with health outcomes, compliance with public health measures, and overall satisfaction with the policy. It is also an evaluation of the impact of decisions on mental health, economic stability and social cohesion.	Challenging to measure all outcomes comprehensively in real-time.	Enhances trust and allows adjustments to improve policy effectiveness.
**PRECIS-2 Tool**	Trial Design	To ensure inclusivity and representativeness in trial populations during health crises.	Broadening the eligibility criteria during a pandemic will ensure that the results can be generalized to a diverse population, as seen in the COVID-19 trial. Rapid enrolment and broad coverage can be achieved through pragmatic recruitment approaches, such as using health systems. These strategies respond to the urgency and inclusiveness that is required in times of health crisis.	Requires expertise in trial design, limiting usability for non-research professionals.	Promotes rapid implementation and relevance to diverse populations.
	Setting and Organization	To mirror typical intervention settings for feasible and realistic implementation.	To reflect typical intervention conditions, pandemic trials should be conducted in real-world settings, such as community health centers or telemedicine platforms. To ensure practical and feasible implementation, they should be organized according to the capacities and constraints of the health system under stress.	Limited feasibility in severely resource-constrained or overwhelmed health systems.	Aligns trial structure with actual healthcare delivery environments.
	Flexibility (Delivery and Adherence)	To evaluate interventions under practical, real-world conditions with variable adherence.	Pandemic interventions need to be adaptable to fluctuating resources and allow for varying delivery, such as remote monitoring or flexible dosing. Pragmatic trials focus on observation of real-world adherence, reflecting how interventions work in usual practice, especially when external factors affect adherence.	May reduce control over intervention fidelity and increase	Ensures applicability to dynamic, resource-

				variability in outcomes.	constrained scenarios.
	Follow-up and Analysis	To reduce healthcare burden and focus on outcomes most relevant to pandemic responses.	Surveillance strategies should use existing health records or patient-reported outcomes during pandemics to reduce the burden on health care systems. Analyses should have an emphasis on real-world relevance, with a focus on critical outcomes such as mortality, hospitalization rates or quality of life, rather than overly precise metrics.	Lower precision in specific contexts may limit granular findings.	Maintains focus on meaningful, actionable outcomes that align with real-world healthcare needs.
**Health Technology Assessment (HTA)**	**Rapid Assessment**	Time-intensive and may lack agility for rapidly changing scenarios.	Pandemic diseases require rapid assessments of diagnostics, treatments, vaccines and digital health solutions, and HTA agencies use accelerated processes which emphasise effectiveness, safety and health system implications. Given the urgency of the situation, real-world evidence, including observational studies and case series, often take the place of long-term clinical trials. These approaches enable timely, informed decisions in crisis situations.	Time-intensive and may lack agility for rapidly changing scenarios.	Enables timely, data-driven decision-making in crisis situations when rapid action is needed.
	**Resource Allocation**	Comprehensive evaluation of cost-effectiveness and feasibility of interventions.	In pandemics, by considering clinical effectiveness alongside economic factors such as cost-effectiveness, budgetary impact and societal value, HTA has a critical role to play in determining the cost-effectiveness of interventions. In addition, particularly when pandemics expose or exacerbate existing health inequalities, HTA can address equity issues and help ensure that technologies and services are distributed equitably to those most in need.	Optimizes resource use and ensures fair distribution of healthcare services, contributing to more equitable healthcare delivery.	Ensures optimal use of limited resources during pandemics by evaluating the cost-effectiveness and feasibility of various interventions.
**SOAR Framework**	Strengths	To identify the internal capabilities that give the organization a competitive advantage.	Strengths represent the internal capabilities that give an organization a competitive advantage. These could include a skilled workforce, cutting-edge technologies or a strong brand reputation. Identifying these strengths helps the organization focus on areas where it can maximize its impact.	Helps the organization focus on maximizing its impact by leveraging its strengths.	May overlook critical challenges and weaknesses if not balanced with other frameworks.
	Opportunities	To identify external factors, such as market trends or technological	Opportunities are external factors that the organization can exploit to achieve its objectives. They may include emerging market trends, technological advances or unmet customer needs. Identifying	Provides a focus for strategic direction, fostering growth, and	Might lead to overestimating the potential if

		advancements, that the organization can capitalize on for growth and innovation.	opportunities enables the organization to focus its strategies on growth and innovation.	improving innovation.	external factors change rapidly.
	Aspirations	To define the long-term vision and motivating goals that guide the strategic direction of the organization.	Aspirations are the long-term goals and visions that drive the strategic direction of an organization. They are a vision of a future state that motivates the team and provides a clear sense of purpose. Aspirations guide the organization in aligning efforts and resources to achieve a shared vision.	Encourages optimism, long-term planning, and alignment of resources to a shared vision.	May lead to unrealistic goals if not grounded in current realities.
	Results	To establish measurable outcomes that track progress toward achieving the organization's goals and objectives.	Results refer to the measurable outcomes that demonstrate progress towards achieving the organization's goals. These can include financial growth, customer satisfaction or market position. Establishing results ensures accountability and helps track the effectiveness of the organization's strategies.	Ensures accountability, helps measure success, and evaluates the effectiveness of strategies.	Can overly focus on short-term metrics, potentially neglecting long-term goals.
**Risk Assessment and Management**	**Risk Identification**	To recognize and prioritize risks to minimize the potential harm to individuals and the health system.	Identifying hazards and exposure risks, such as pathogen characteristics and high-risk environments, is part of pandemic risk assessment and management. Impact and vulnerability assessments and the implementation of preventive measures, as well as continuous monitoring and adaptive responses, are part of effective assessment and mitigation strategies.	Effective at pinpointing high-risk areas for targeted intervention.	Depends on accurate data, which may be scarce in early pandemic stages.
	**Risk Analysis**	To estimate the severity and probability of risks and their consequences.	Dose-response assessments, which evaluate how pathogens affect individuals based on exposure levels and severity, are part of risk analysis during a pandemic. Risk characterization estimates the likelihood of adverse outcomes, including secondary effects such as economic or social disruption, by combining hazard identification and exposure assessment.	Helps estimate the severity and probability of various outcomes.	Data on exposure levels and effects may be insufficient, especially in early stages of a pandemic.
	**Risk Management**	To mitigate the impact of risks through effective interventions and resource distribution.	There are several key strategies for managing risk during a pandemic. Prevention strategies are implemented based on predictive models and past experience, such as social distancing and hygiene practices. As seen in the response to H1N1 influenza, control measures such as quarantine, isolation and contact tracing help to contain the spread. Resource allocation optimizes the public health response and minimizes its impact by ensuring that critical resources, such as vaccines and PPE, are distributed to areas or populations at highest risk.	Provides structured strategies for managing pandemic risks.	May struggle in the face of limited resources during early pandemic stages.

	**Risk Communication**	To ensure informed decision-making and encourage compliance with public health measures.	To guide public behaviour and ensure compliance with public health measures, risk communication during a pandemic is essential. As demonstrated by effective communication strategies during COVID-19, public information must be clear, consistent and timely to inform people about risks, preventive measures and updates. Stakeholder engagement ensures that interventions are context-specific and effectively implemented in local settings by working with community leaders, health care providers and policy makers.	Facilitates public compliance and ensures tailored interventions.	May face challenges in ensuring accurate messaging and engagement across diverse communities.
	**Risk Monitoring and Review**	To track the progression of the pandemic and assess the effectiveness of strategies.	Monitoring and reviewing risks during a pandemic involve continuous monitoring and evaluation to adjust strategies as needed. To support timely decision-making, surveillance systems provide real-time data on disease progression, mutations and the effectiveness of control measures. Regular evaluation ensures that risk management strategies remain effective and that necessary adjustments are made based on new information or evolving conditions. This allows for dynamic and responsive approaches.	Ensures timely adaptation of strategies to changing circumstances.	Can be overwhelmed by the scale of data and rapid developments in fast-moving pandemics.
**DECIDE Framework**	Define the Problem	To clearly articulate the issue, its scope, and its impact, ensuring that the problem is well-understood.	Clearly articulate the problem. This involves understanding what the problem is, how it's defined, and how it's affected. In a business context, that could be defining why a product doesn't sell as well as expected.	May not fully capture all underlying causes, especially in complex or ambiguous situations.	Ensures a focused approach by identifying the core issue, preventing misdirection.
	Establish the Criteria	To set clear standards for evaluating potential solutions, aligning them with the goals and objectives.	Decide on the factors that will be used in the evaluation of the solutions. These might include cost, time, risk, effectiveness, aligning with organizational values or satisfying stakeholders. In a health care context, the criteria might include patient outcomes, compliance with regulatory requirements, or cost effectiveness.	Criteria selection may be subjective, influenced by personal biases or insufficient data.	Provides a measurable framework to assess alternatives, making the decision-making process transparent.
	Consider the Alternatives	To generate a variety of potential solutions or strategies, encouraging creativity and thorough exploration.	Brainstorming or researching possible solutions or strategies. This step is an exercise in creativity and should look beyond obvious solutions to innovative or less conventional approaches. Alternatives could range from regulatory measures to incentive schemes for the uptake of green technologies in environmental policy, for example.	Time-consuming if many alternatives are considered, and can lead to analysis paralysis.	Encourages innovation and broad thinking, exploring unconventional or creative solutions.

	Identify the Best Alternative	To evaluate each option based on the established criteria, selecting the most viable solution.	Evaluate each alternative against the established criteria. This may involve a qualitative analysis, where the input of stakeholders is crucial, or it may involve quantitative methods such as cost-benefit analysis. To compare options systematically, decision tools such as decision matrices can be used.	The evaluation process can be overly simplified if criteria are not comprehensive or quantitative methods are misused.	Systematic comparison using criteria helps avoid bias and makes decisions more grounded and defensible.
	Develop and Implement a Plan	To create a structured plan for action, including timelines, resources, and responsibilities.	Draw up a detailed plan for the implementation of the chosen alternative. This will include the setting of timelines, the allocation of resources, the definition of responsibilities and the outlining of steps. In project management terms, this would be the creation of Gantt charts or task lists.	Implementation may face resistance or practical challenges not anticipated during the planning stage.	Provides a clear roadmap, ensuring the chosen alternative is put into action effectively and efficiently.
	Evaluate and Monitor the Solution	To assess the solution's performance and outcomes, adjusting the plan as necessary based on feedback and data.	After implementation, to assess whether the solution is meeting expectations, monitor the results against the defined criteria. If the solution isn't working as expected, adjustments may need to be made or earlier steps may need to be revisited.	Ongoing evaluation may be resource-intensive, and may require adjustments that could disrupt operations.	Allows continuous improvement, providing feedback to adapt strategies to new conditions or challenges.
**AI-Driven Decision Support Systems**	Data Collection and Integration	To collect and integrate comprehensive data from diverse sources for a complete view of the pandemic.	To provide a comprehensive view of the pandemic, AI-DSS collects and integrates data from multiple sources, including health records, social media and mobility data. Real-time data processing provides critical insight into disease progression and containment measures, ensuring the system is always up to date.	Data accuracy and availability may be a challenge, especially in the early stages of a pandemic.	Provides real-time insights into disease progression and response effectiveness, ensuring timely decision-making.
	Data Analysis and Modeling	To use predictive models and anomaly detection to forecast future events and identify emerging threats.	AI supports proactive response strategies by using predictive models such as SEIR to forecast disease spread and resource requirements. By analyzing non-traditional data patterns, anomaly detection can identify emerging outbreaks for early intervention	Models may be inaccurate if data quality is poor or if assumptions do not hold in real-world scenarios.	Allows for proactive planning and early intervention by predicting

					disease spread and resource needs.
	Decision Support Tools	To help decision-makers simulate various intervention strategies and optimize resource allocation.	AI-based scenario planning helps decision-makers understand potential outcomes by simulating different intervention strategies. Resource optimization algorithms ensure that resources are directed to areas of greatest need by efficiently allocating medical supplies and personnel.	Over-reliance on AI-generated recommendations without human judgment may lead to suboptimal decisions.	Enables efficient resource use and informed decisions to minimize the pandemic's impact.
	User Interfaces and Dashboards	To present real-time data in a visually intuitive way that aids decision-making at various levels.	Intuitive dashboards help public health officials and healthcare providers make real-time decisions by visualizing key metrics such as disease spread and resource use. Users can tailor responses by adjusting variables and analyzing potential impacts using interactive decision support tools.	High complexity and overload of information can confuse users, especially if dashboards are not tailored to specific needs.	Easy-to-understand visualization tools help guide informed decisions based on real-time metrics and predictions.
	Communication and Dissemination	To deliver clear, consistent, and timely messages to the public and stakeholders.	AI generation of automated reports for timely and transparent health updates. Natural language processing optimizes public health messages to ensure clarity and sensitivity to different cultures.	Language or cultural barriers may limit message effectiveness in diverse populations.	Ensures effective communication of health risks and interventions, helping to maintain public trust and compliance.
	Feedback Loop and Learning	To continuously improve AI predictions and adapt the system based on real-time outcomes and user feedback.	Through continuous learning, AI systems are able to refine their models and predictions based on the results of past decisions, thereby improving their accuracy over time. Incorporating user feedback ensures that AI continues to adapt to real needs and remains effective in guiding pandemic response efforts.	Feedback loops may take time to show results, and user feedback may be inconsistent or difficult to quantify.	Enables adaptive learning, ensuring that the system improves with each iteration, making the decision

					support more accurate.
